# Morphometric characterisation of human tracheas: focus on cartilaginous ring variation

**DOI:** 10.1186/s13104-018-3123-1

**Published:** 2018-01-16

**Authors:** Y. Premakumar, M. F. Griffin, M. Szarko

**Affiliations:** 1 Anatomical Sciences, St. George’s University of London, Cranmer Terrace, London, SW17 0RE UK; 2 UCL Division of Surgery & Interventional Science, Centre for Nanotechnology & Regenerative Medicine, University College London, London, UK

**Keywords:** C-shaped ring, Human trachea, Tracheal anatomical variation, Tracheal anatomy, Tracheal ring

## Abstract

**Purpose:**

Details regarding tracheal anatomy are currently lacking, with existing literature focussing mainly on the cricoid-tracheal region or the carina. External gross anatomy and internal morphology throughout the entire trachea is important for normal physiological functioning and various clinical applications such as designs for tracheal implants or endotracheal devices.

**Objective:**

To determine quantitative and qualitative characteristics of gross tracheal and individual tracheal ring anatomy.

**Method:**

10 tracheas were harvested from formaldehyde-fixed cadavers. Tracheal length, height and inter-ring distance were measured from complete tracheas. Individual rings were excised and the following measurements were taken at three points on the ring: thickness, width, and antero-posterior (A-P) length.

**Results:**

The average tracheal length was 10.38 ± 0.85 cm with a mean of 19 ± 3 rings per trachea. The average width and A-P diameter of tracheal lumens were 17.31 ± 2.57 and 17.27 ± 2.56 mm, with a width-AP ratio of 1.00 (‘C’ shaped ring). The A-P diameter shows a trend of narrowing slightly from the upper 1/3 to the lower 1/3 of the trachea. While majority of tracheal rings consisted of the expected ‘C’ shape, more than 41% of the 147 counted rings consisted of abnormally shaped rings which were further analysed.

**Conclusion:**

This study provides further details regarding tracheal anatomy which will be useful for implant design. Of interest for anatomists, is the marked variability in tracheal ring morphology which could be further characterised in larger studies.

## Introduction

**Fig. 1:**
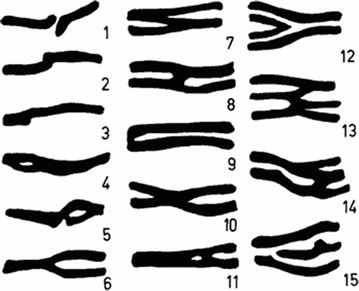
A diagrammatic representation of the various single and compound rings which were encountered in the lower part of the trachea in a human adult. The following are the assigned titles: (1) interrupted, (2) S-shaped, (3) L-shaped, (4) and (5) fenestrated, (6) Y-shaped or forked, (8) H-shaped, (9) U-shaped, (10) X-shaped, (11) A-shaped, (12) V-shaped, (13) and (14) M or W-shaped, (15) incomplete (Reproduced from Vanpeperstraete [1])

In the late 1800 s few textbooks showed shapes of the tracheal rings, of which, the description given by Ph. Sappey in 1874 was the most detailed. It translates as follows: “Sometimes however they [tracheal rings] are unlike the normal and take on varied shapes…one finally observes, that, compared to each-other, not two of them look exactly alike”. After conducting a very thorough literature search [1] concluded that a multitude of shapes exist in the lower trachea (see Fig. 1). However, while the shapes were clearly outlined, the frequency of ring appearance and the presence of these ring variations in other parts of the trachea were not studied due to limited parts of trachea available.

A review of the current literature regarding the morphology of trachea cartilage shows little to no research further exploring this frontal-plane variation in ring shape. Chunder et al. [2] is the most recent and the only study, the authors could find, to note ring variation in the axial plane. This neglected area of study is important for anatomical understanding as well as clinical practice. The effect of the presence or absence of cartilage in bronchial and pulmonary conditions is important since obstruction of airways depends on the trachea’s rigidity, which in turn is partially determined by the shape of its cartilaginous rings [1]. Absent or deformed cartilage rings will result in poor structural support in those regions [3].

Morphology of cartilaginous rings is equally important for airflow. The trachea is significant in its role of being one of the most immediate passages air will flow through to reach pulmonary parenchyma. Narrowing of the airway radius will have an exponential effect on laminar airflow, as Poiseuille’s equation states [3]. Absent or deformed cartilage rings will result in poor structural support in those regions.

There are many congenital tracheal conditions, including tracheal webs and stenosis, tracheomalacia, tracheal cleft and tracheal atresia, leading to potentially fatal consequences due to hypoxaemia [3]. The numerous congenital tracheal malformations along with severe tracheal neoplasms infiltrating adult airways are some of the most significant indications for tracheal implants. Tracheal implants are a relatively new form of treatment allowing for customisable reproducible airway creation without relying on cadaveric donations or temporary stents. Where an anatomically correct but non-functional airway exists, for example tracheobronchomalacia, it is possible to recreate implants based on computed-tomography (CT) imaging. However, where there are anatomical defects in the trachea, for example severe stenosis or carcinoma, implants will rely less on the patient’s original airway. This is where accurate characterisation of the airway becomes important.

For a clinical focus, two aspects of tracheal anatomy are important: intraluminal dimensions and gross external anatomy. The intraluminal dimensions are key for endotracheal procedures such as intubation, stenting, and surgery; while the gross anatomy, referring to the entire trachea and individual rings, is important for implant design.

It has been reported that the tracheal length is approximately 11 cm and it has cartilaginous rings anteriorly and laterally with the trachealis muscle posteriorly. There are conflicting reports regarding the cross-sectional shapes of tracheal rings, with different sources stating ‘D’, ‘C’, or ‘U’ shapes. Tracheal cartilage rings consist of single rings with 1 mm thickness and 4 mm depth on average. Though it should be noted that the shape of the adult trachea varies with disease due to intraluminal pressure changes (e.g. chronic obstructive pulmonary disease) [4].

In terms of gross tracheal anatomy, recent literature contains few papers who have aimed to characterise gross tracheal anatomy. Kamel et al. [5] characterised in situ tracheal anatomy by analysing chest CT imaging of 60 patients and 10 cadavers. Another study characterised some aspects of tracheal anatomy on 61 autopsy material but focused mainly on cricoid anatomy [6]. Neither of these studies addressed the details of tracheal gross anatomy. These tracheal details, such as individual ring characteristics, are pertinent because implants aim to mirror native organ function.

In terms of internal tracheal anatomy, antero-posterior (A-P) and transverse widths have been extensively studied [2] however there has been little work done on the frontal shapes of the rings. The cross-sectional shapes are important considerations for ensuring accurate fits of endotracheal devices while the frontal shapes will ensure accuracy in synthetic implants.

Overall, the intraluminal dimensions have been previously studied, however there is a lack of information regarding morphological details of the gross trachea and individual rings’ appearance. This study aims to simply characterise the gross anatomy and intraluminal dimensions of individual cartilaginous rings in the human trachea.

## Methods

### Harvesting tracheas

10 formaldehyde-fixed cadavers (8 male, 2 female, age range 70–96 years) were obtained and utilised in accordance with the Human Tissue Act (2004). They were embalmed with 10% formaldehyde, 10% polyethylene glycol, 5% phenol and 75% ethanol. The trachea was exposed in situ from the cricoid to the main bronchi. This required extensive dissection to clear away bony structures and visceral tissue. The trachea was separated from the cricoid by cutting through the crico-tracheal ligament, and separated from the main bronchi by making incisions 3 cm distal to the carina.

### Measurements and analysis

**Fig. 2:**
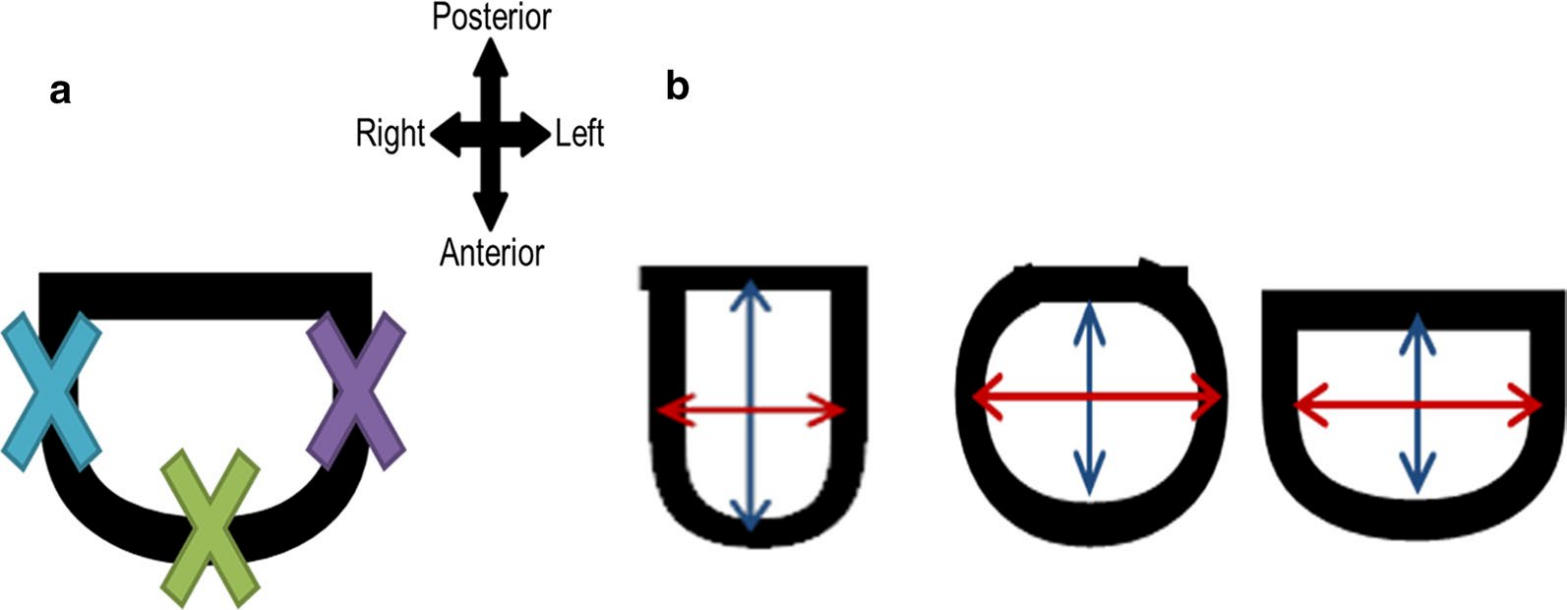
**a** Diagram illustrating where the three points of measurement for each ring were taken for thickness and height. The right end (blue) and left end (purple) measurements are taken 6 mm from the cartilage-trachealis border. **b** Diagram illustrating width and A-P measurements, and the associated cross-sectional ring shapes (U, C, D). Blue line: A-P diameter. Red line: width

The harvested tracheas had the following measurements taken by a digital vernier caliper (accuracy ± 0.01 mm): trachea length (superior border of 1st tracheal ring to inferior border of carina), height (superior border of ring to inferior border of individual ring), and inter-ring distance (inferior border of proximal ring to the superior border of the distal ring). Due to a mix of single rings and abnormally-shaped rings, it was difficult to count the number of rings. The abnormal rings were counted as one ring for counting purposes, even if they had an obvious appearance of multiple ring fusion. The following measurements were taken before excision of individual rings: thickness, width of entire ring, A-P length of entire ring. Height and thickness measurements were taken at three points including the respective ends of the ring to see if there is a difference in the cartilage attached to the trachealis muscle compared to cartilage that is not placed under similar forces (Fig. 2a).

A width-AP ratio was calculated and assigned an associated cross-sectional ring shape: ‘U’ if < 1, ‘C’ if 1, ‘D’ if > 1 (Fig. 2b).

Diagrams of every trachea’s rings were drawn with specific categories assigned afterwards based on repeated shapes for classification and analysis purposes (see Table 1).

**Table 1:** Diagrams and brief descriptions of the shapes identified for tracheal ring classification

Assigned code	Tracheal ring shape	Description of ring
A		A singular band of tracheal cartilage resembling a ring throughout with parallel borders
B		A straight band of tracheal cartilage on the left half of the ring, and a split into two distinct bands at the right end
C		A straight band of tracheal cartilage on the right half of the ring, and a split into two distinct bands at the left end
D		Two singular rings of tracheal cartilage connected by a band of cartilage directed infero-medially from the right end of the proximal ring
E		Two singular rings of tracheal cartilage connected by a band of cartilage directed infero-medially from the left end of the proximal ring
F		A thick ring of tracheal cartilage with two distinct bands on both right and left ends
G		A thick ring of tracheal cartilage with a slit in the centre
H		A band of tracheal cartilage that resembles an upright pentagon in shape with an apex in the centre of the superior border
I		A band of tracheal cartilage that resembles an inverted pentagon in shape with an apex in the centre of the inferior border
J		A triangular piece of cartilage on the left side that did not extend beyond the tracheal midline. Connective tissue occupied the rest of the layer
K		A triangular piece of tracheal cartilage on the right side that did not extend beyond the tracheal midline. Connective tissue occupied the rest of the layer
L	Other	A ring shape which does not conform to the aforementioned descriptions or is a combination of one or more of the ring types

## Results

### Gross tracheal dimensions

The average tracheal length was 10.38 ± 0.85 cm with a mean of 19 ± 3 rings per trachea. The overall average thickness and height for every ring was 1.83 ± 0.36 and 4.53 ± 1.62 mm, respectively. The mean inter-ring distance of the upper 1/3 (1.52 ± 0.61 mm), middle 1/3 (1.23 ± 0.38 mm), and lower 1/3 trachea (1.35 ± 0.53 mm) was 1.38 ± 0.56 mm.

### Intraluminal dimensions

The average width and A-P diameter of tracheal lumens were 17.31 ± 2.57 and 17.27 ± 2.56 mm, with a width-AP ratio of 1.00 (‘C’ shaped ring). On average, the intraluminal width is greatest in the middle 1/3 of the trachea while the A-P diameter shows a trend of narrowing slightly from the upper 1/3 to the lower 1/3 of the trachea. The width-AP ratios illustrate this relationship better: 0.98 (upper 1/3), 1.01 (middle 1/3), 1.00 (lower 1/3).

### Individual ring shapes

**Fig. 3:**
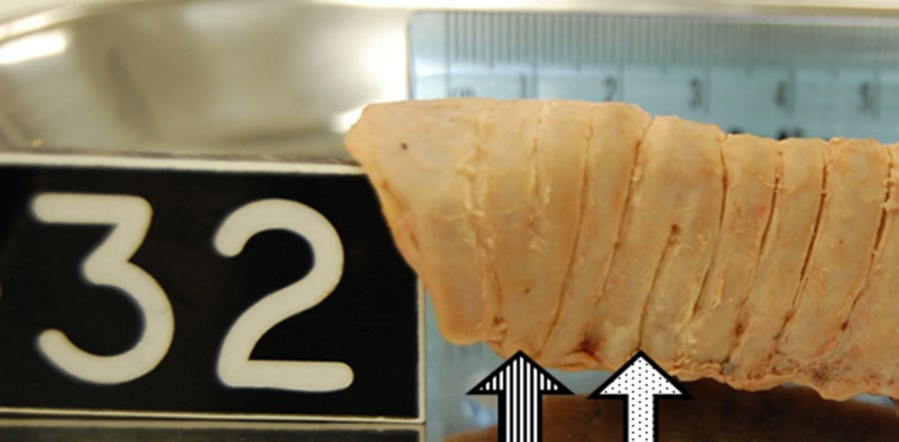
Parasagittal view of Cadaver 32’s upper trachea from the right side. Striped arrow highlights ‘G’ and dotted arrow highlights ‘B’ ring type. Ruler (cm) is in the present for reference

While majority of tracheal rings consisted of the expected ‘C’ shape, more than 41% of the rings consisted of abnormal rings (see Table 2, Fig. 3).

**Table 2:** Total number of ring types identified and the distribution based on their location on the trachea

Ring type	Absolute # of rings (% of the total # of rings)	% of specific ring type found		
		Upper 1/3 of trachea	Middle 1/3 of trachea	Lower 1/3 of trachea
A	86 (58)	34	49	17
B	13 (9)	31	31	38
C	8 (5)	38	12	50
D	4 (3)	25	50	25
E	2 (1)	50	50	0
F	1 (1)	0	100	0
G	6 (4)	83	17	0
H	3 (2)	100	0	0
I	6 (4)	0	0	100
J	3 (2)	67	33	0
K	1 (1)	0	0	100
L	14 (10)	50	36	14
Total	147 (100)

### Distribution of individual ring shapes

There was variable distribution of ring shapes throughout the tracheal regions (see Table 2). Asymmetrical ring patterns were found to have the cartilage abnormality on the right-side more often than the left (B, D). The distinct presence of the shield-like ‘H’ and ‘I’ ring in the upper and lower 1/3 as the 1st and last ring has been noted before (1).

Asymmetrical ring patterns were found to have the cartilage abnormality on the right-side more often than the left (B, D). ‘I’ ring type was only found in the lower 1/3 of the trachea, always as the last ring before the carina. Whereas ‘H’ ring type was found only in the upper 1/3 of the trachea, always as the 1st ring of the trachea. The ‘B’ and ‘C’, similar variations, were present more in the lower 1/3 of the trachea.

## Discussion

There are conflicting reports regarding what constitutes a normal trachea length with some studies reporting a range of 10–13 cm whereas one study reported a normal length as approximately 17.01 ± 1.28 cm, and according to them, ‘short trachea’ was reported to be when the tracheal length was 15 cm or less [7–9]. Based on studies within the past two decades, the average trachea length range is approximately 9–11 cm [2, 5, 6]. This study found that the average tracheal length was 10.38 ± 0.85 cm. It should be noted that the methods used kept tissue shrinkage to a minimum. The reduction in tracheal length may be due to the elderly populations studied in all cases and the age-related increase in inter-ring fibrous tissue contraction [2]. Another consideration is how there are many congenital conditions associated with ‘short trachea’ [10], however the complete medical history of the cadavers was unattainable therefore it was not possible to see whether this was a confounding factor.

Secondly, in order to create accurate tracheal implant ring appearances, overall average thickness and height measurements could be used throughout the ring since there was no significant difference between tracheal regions (upper, middle, lower 1/3 of the trachea) and between various points on an individual ring. It was assumed there would be a difference between the cartilage at the centre of tracheal rings compared to the cartilage at the ends of the rings attached to trachealis muscle based on cartilage adaptation to environmental forces [11]. Of note was the greatest inter-ring distance found at the upper 1/3 of the trachea (1.52 ± 0.61 mm). This increase in connective tissue area between rings may be due to the need for increased tracheal flexibility in the highly mobile larynx compared to tracheal regions that sit in the mediastinum. Also, after amalgamating the similar results from Kamel et al. [5] and this study, the number of cartilaginous rings to incorporate into synthetic tracheas ranges from 16 to 19 if it is to mimic native tracheas.

In terms of designing the implant to mimic native trachea lumens, the width and A-P diameter should be modelled using the averages of 17.31 ± 2.57 and 17.27 ± 2.56 mm to give cross-sectional ‘C’ shaped rings (a width-AP ratio of 1.00) for majority of rings. Majority of tracheal rings were identified as ‘C’ shaped but there were differences in shapes based on location. More specifically, the average 0.98 width-AP ratio for the upper 1/3 of the trachea indicates a larger number of ‘U’ shaped rings, while the average ratio of 1.01 in the middle 1/3 would infer a larger presence of ‘D’ tracheal rings, and 1.00 in the lower 1/3 would suggest the presence of ‘C’ tracheal rings. There are said to be biomechanical implications for these non-linear shapes in maintaining an airway, however the exact effect is poorly studied and thus no conclusions can be drawn [12, 13].

So far, the following initial conclusions from this are: 16–19 individual rings should be designed to have a uniform thickness and height, and similar width and A-P lengths to produce ‘C’ shaped rings in the axial plane. As this is a small sample size and foundation study, further studies are needed with more diverse samples (size and gender).

After a survey of the literature, this study stands as the first to identify ring variation. Majority of anatomy resources state that tracheal rings exist as single parallel bands, however this has proven to be incorrect. Previous studies have identified various shapes but there seems to be a neglect of attention on this detail in publications. As mentioned previously, there are theoretical biomechanical implications for various tracheal ring shapes however the practical consequences of these are poorly understood [12, 13]. It has been postulated that oblique bands of cartilage help tracheal adaption to a variety of motion that it experiences under constantly changing intrathoracic pressures. Obliquely angled cartilage and compound rings consisting of fused rings may help maintain the necessary structural support for maintaining open airways [12]. Most importantly, this acknowledgement and characterisation of ring shapes could serve as a reference for future studies in an attempt to expand the existing knowledge base concerning human tracheal anatomy.

Even though the small sample size greatly limits this study and its findings for clinical applications, the identification of tracheal ring variations is important in the study of trachea anatomy. Foci for future work could include identifying biomechanical properties and expanding tracheal anatomy characterisation to a paediatric population.

## Conclusion

In summary, there was no obvious difference in intra- or inter-tracheal ring cartilage thickness and height, there is a correlation between tracheal regions and the cross-sectional shape of the ring, and there is a significant range of frontal-plane tracheal ring shapes. Most importantly, this study highlights and further characterises the different ring shapes within tracheas. This variety found within the trachea is important theoretically for educational purposes as well as multiple clinical applications.
